# Solving a Migration Riddle Using Isoscapes: House Martins from a Dutch Village Winter over West Africa

**DOI:** 10.1371/journal.pone.0045005

**Published:** 2012-09-21

**Authors:** Keith A. Hobson, Steven L. Van Wilgenburg, Theunis Piersma, Leonard I. Wassenaar

**Affiliations:** 1 Environment Canada, Saskatoon, Saskatchewan, Canada; 2 Centre for Ecological and Evolutionary Studies, University of Groningen, Groningen, Groningen, The Netherlands; 3 Department of Marine Ecology, Royal Netherlands Institute for Sea Research, Den Burg, Texel, The Netherlands; University of Bern, Switzerland

## Abstract

**Background:**

The ability to connect breeding, stopover and wintering locations of populations of migratory birds greatly enhances our understanding of the phenomenon of migration and improves our chances of effectively conserving these species. Among Palearctic-Afrotropical migratory species, aerial insectivores like the house martin (*Delichon urbicum*) are sensitive to factors influencing the availability of flying insects, and have declined in recent decades. The strict aerial behaviour of martins severely limits ring recoveries on wintering grounds and so there is a dearth of information on where European breeding populations over-winter in Africa, and the relative effects of population regulation on breeding vs. wintering grounds. We used a newly developed multi-isotope (*δ* ^2^H, *δ* ^13^C, *δ* ^15^N) feather isoscape for Africa together with inferences from summarized ring return data based on longitude, to assign winter origins to birds captured at a breeding colony in The Netherlands.

**Principal Findings:**

Based on isotopic analyses of winter-grown martin feathers, we used a likelihood-based assignment approach to describe potential wintering locations where molt occurred of individual house martins from a Dutch colony by assigning them to four potential isotopically distinct clusters in Africa. We found the overwhelming majority of Dutch martins were assigned to a geographical cluster associated with West Africa.

**Conclusions/Significance:**

The existence of strong isotopic gradients and patterns in African foodwebs that support migratory wildlife allows for the spatial assignment of tissues grown there. The assignment of Dutch house martins to wintering grounds primarily in West Africa was in strong agreement with independent and indirect methods used to infer winter origins of this species based on the association between the Normalized Difference Vegetation Index (NDVI) in Africa and population patterns in Italy and the United Kingdom. These confirmatory data-sets underscore the importance of suitable habitats in West Africa to the conservation of migratory aerial insectivores and other species.

## Introduction

House martins (*Delichon urbicum*) are among the most common migratory aerial insectivores in the Old World [1], and among the most mysterious with regard to their winter quarters. Unlike the closely related barn swallow (*Hirundo rustica*), house martins rarely yield recoveries away from their European breeding grounds [2], [3] despite being widely ringed; nor are they frequently observed in Africa (e.g. [4]). Although the suggestion by Carl Linneaus [5] in 1757 (in his academic thesis *Migrationes Avium*) that swallows and martins hibernate at the bottom of lakes was officially rejected by the Swedish Academy of Sciences in 1854, the wintering distribution of house martins has remained enigmatic [6].

In the absence of wing molt in martins on the breeding grounds, and with molt unlikely to happen during migration, feather replacement takes place at the overwintering sites [7], [8], [9]. We conducted a preliminary test of this idea with a comparison of the isotopic composition (*δ* ^2^H, *δ* ^13^C and *δ* ^15^N) of martin feathers between juvenile and adult birds captured at a single breeding site in The Netherlands over six years. We then used the triple isotopic (*δ* ^2^H, *δ* ^13^C and *δ* ^15^N) composition of primary feathers of adult martins returning to their breeding grounds in The Netherlands to provide a first approximation of the most likely regions of Africa in which these feathers were grown by assigning them to isotopic “clusters” in Africa [10]. Finally, we contrast the results of multi-isotope assignment with geographic assignments based upon *δ* ^2^H alone.

## Methods

### Ethics Statement

This study was conducted under permits issued by the Dutch bird ringing centre Vogeltrekstation-Arnhem.

### Samples

House martins were captured at a colony in the village of Gaast (53°01′N, 05°24′E), The Netherlands, from June-August 2005–2010. We captured birds using mist-nets in late afternoon and early evening before nightfall, with small nets at the entrance of individual nests during daytime, by open bags in the case of birds emerging at dawn by paper-plugging of nest entrances. To minimize disturbance, we did not attempt to capture birds until their breeding season was well underway. All birds were processed (ringed, measured and a small blood sample taken for sexing; [11]) and released as soon as possible, with a median holding time of ∼30 min. We cut the tips (∼4 mm) of the innermost primaries (P1) of both wings, and stored the feather (vane) material in paper envelopes until they were processed for isotope assays.

### Stable Isotope Analyses

All feathers were cleaned of surface oils in 2∶1 chloroform:methanol solvent rinse and prepared for *δ* ^2^H, *δ* ^13^C and *δ* ^15^N analysis at the Stable Isotope Laboratory of Environment Canada, Saskatoon, Canada. The non- exchangeable hydrogen of feathers was determined using the method described by [12] and using two calibrated keratin hydrogen-isotope reference materials. Hydrogen isotopic measurements were performed on H_2_ gas derived from high-temperature (1350°C) flash pyrolysis of 350±10 ug feather subsamples and keratin standards using continuous-flow isotope-ratio mass spectrometry. Measurement of the two keratin laboratory reference materials (CBS, KHS) corrected for linear instrumental drift were both accurate and precise with typical mean *δ* ^2^H ± SD values of –197±0.79 *‰* (*n* = 5) and −54.1±0.33 *‰* (*n* = 5), respectively. All results are reported for non-exchangeable H expressed in the typical delta notation, in units of per mil (*‰*), and normalized on the Vienna Standard Mean Ocean Water – Standard Light Antarctic Precipitation (VSMOW-SLAP) standard scale.

For *δ* ^13^C and *δ* ^15^N analyses, between 0.5 and 1.0 mg of feather material was combusted online using a Eurovector 3000 (Milan, Italy - www.eurovector.it) elemental analyzer. The resulting CO_2_ and N_2_ was separated by Gas Chromatograph (GC) and introduced into a Nu Horizon (Nu Instruments, Wrexham, UK - www.nu-ins.com) triple-collector isotope-ratio mass-spectrometer via an open split and compared to a pure CO_2_ or N_2_ reference gas. Stable nitrogen (^15^N/^14^N) and carbon (^13^C/^12^C) isotope ratios were expressed in delta (*δ*) notation, as parts per thousand (*‰*) deviation from the primary standards: atmospheric nitrogen and VPDB (Vienna Pee Dee Belemnite carbonate) standards, respectively. Using previously calibrated internal laboratory C and N standards (powdered keratin and gelatin), within runs, precisions for *δ* ^15^N and *δ* ^13^C were better than ±0.15 *‰*.

### Statistical Analysis

We compared *δ* ^2^H, *δ* ^13^C, and *δ* ^15^N in feathers of adult versus juvenile house martins using a two sample t-test. Data from adult house martins were subsequently analyzed using linear mixed effects models (LME) [13]. We used LME with ring number treated as a random effect to control for repeated measurements made on individuals between years, and tested for inter-annual variation by treating year as a fixed-effect factor. We did not consider sex as a candidate variable since a preliminary examination of the data showed no underlying pattern with sex for any isotope. Post-hoc differences between years were tested with Tukey’s tests implemented the R package multcomp [14].

### Geographic Assignment

We had data for three isotopes (*δ* ^2^H, *δ* ^13^C, and *δ* ^15^N) for samples collected from 2007–2010. However, the 2009–2010 overwintering period appeared climatically anomalous within Africa based on examination of climate indices (Indian Ocean Dipole Mode Index, and Multivariate ENSO); thus we expected that samples collected in 2010 would not fit the long-term isoscapes (particularly *δ* ^2^H) well. As a result, we omitted data from 42 birds for which we had data on all three isotopes from 2010. Data for *δ* ^13^C and *δ* ^15^N were not available for samples collected in 2005 and 2006. Thus, we used data from 171 house martins sampled during 2007–2009 in assignment of individuals to isotopic clusters’ representing isotopically distinct regions of Africa [10]. In brief, [10] used multivariate cluster analysis of previously published plant *δ* ^13^C [15], and *δ* ^15^N [16] isoscapes and the long-term hydrologic *δ* ^2^H [17] isoscapes of Africa to derive four isotopically distinct regions of Africa. The *δ* ^13^C and *δ* ^15^N isoscapes were converted to feather isoscapes by assuming isotopic discrimination of +2 *‰* and +5 *‰* respectively from diet to tissue [10]; whereas the *δ* ^2^H isoscape of [17] was calibrated using data from known-source birds using the following equation (*δ* ^2^H_f_ = −6.77+1.42 *δ* ^2^H), (for further details see Text S1).

We used a likelihood-based assignment test to determine which isotopic cluster represented the most probable origin for a given feather sample, given the regional mean expected values for the three isotopes and the covariance among isotopes. The likelihood that an isotopic cluster represented the origin for a sample was estimated using a multivariate normal probability density function [18]. Mean expected isotope values for each of the isotopic clusters within Africa (Table S1) were determined by summarizing cells within each isoscape falling within each isotopic cluster using an ArcGIS zonal statistics query. We estimated the covariance among all three isotopes in our sample of house martins using the ‘mvnmle’ package within R 2.13.0 [19]. Using expected feather isotope means from the isoscapes and the covariance matrix between isotopes defined from our feather samples, we estimated the multivariate normal probability densities associated with each potential source population using the mvtnorm package [20], [21] in R 2.13.0 [19]. Probability densities were subsequently normalized (i.e. divided by the sum of the probabilities). To fully account for all sources of isotopic variance, we repeated this step 1000 times, in each case simulating data with the observed feather *δ* ^2^H, *δ* ^13^C, and *δ* ^15^N values for each individual representing the mean of the normally distributed simulation.

Within a given simulation, we assigned feather samples to an isotopic cluster by associating it with the cluster for which the highest likelihood was found. However, some samples were occasionally assigned to different clusters between simulations. Thus, we report mean number of birds (± SD) assigned to a given isotopic cluster across simulations. In order to further interpret our geographic assignments based upon stable isotope assays we also examined ring-recovery data for trans-Saharan house martin recoveries reported in [22]. Based upon inspection of recovery depictions in [3], we modeled recovery longitude as a function of ringing longitude using linear regression.

## Results

As expected, all feathers grown by hatching-year birds in The Netherlands differed isotopically from adults that grew their feathers in Africa, being comparatively depleted in ^13^C and ^2^H and enriched in ^15^N (*δ* ^2^H: HY = −89.4±9.8 *‰*, n = 81; Ad = −46.3±13.4 *‰*, t = 26.8, df = 1,41, p<0.0001; *δ* ^13^C: HY = −21.4±1.3 *‰*, n = 48; Ad = −16.7±1.3 *‰*, n = 212, t = 22.7, p<0.0001; *δ* ^15^N: HY = 16.1±0.9 *‰*, n = 48; Ad = 10.3±0.9 *‰*, n = 212, t = 41.2, df = 1,258, p<0.0001). The high HY *δ* ^15^N values reflect the strong influence of the agricultural landscape of the Netherlands.

**Figure 1:**
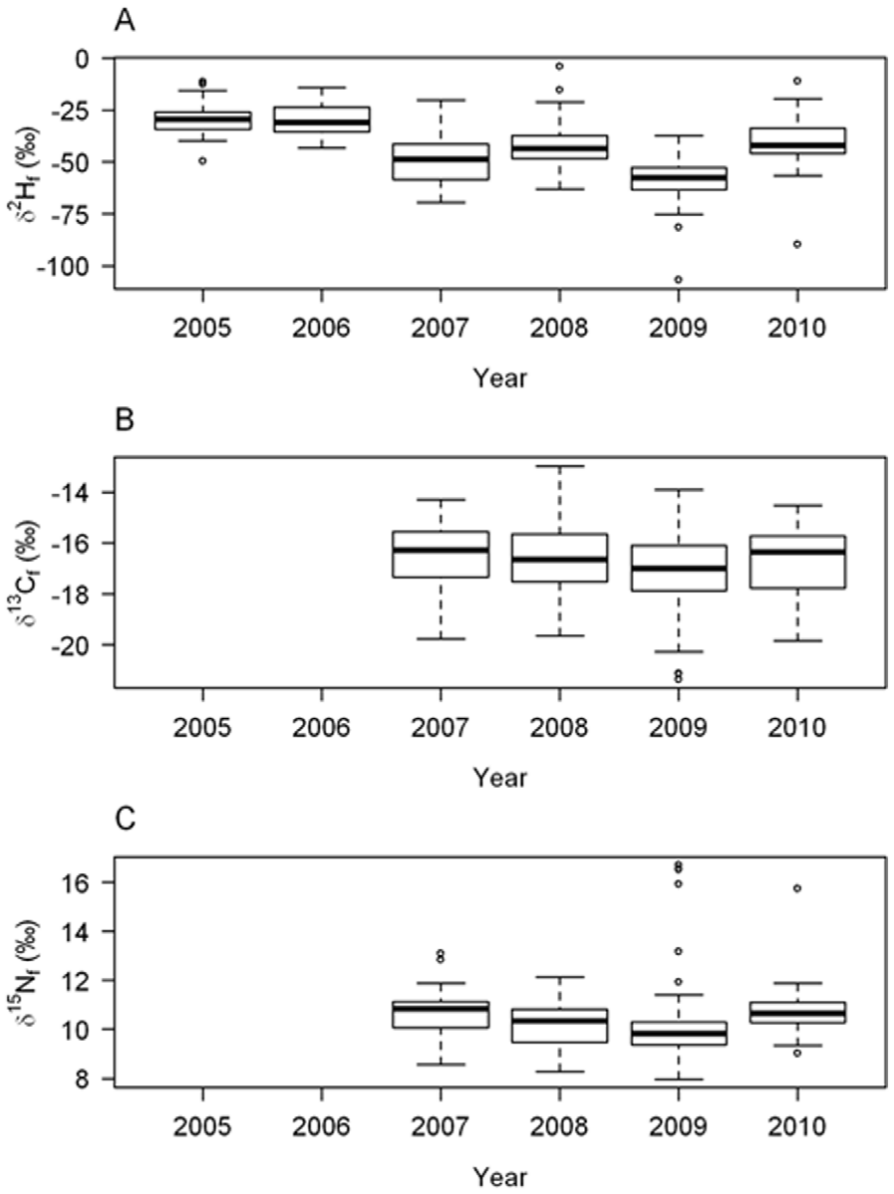
Variation in the isotopic composition of Aftrotropical grown house martin feathers from birds breeding in The Netherlands from 2005 to 2010 for A) *δ* ^2^H, B) *δ* ^13^C, C) *δ* ^15^N.

Among adults, we found substantial inter-annual variation in feather *δ* ^2^H values (Table 1, Figure 1A). In general, feather *δ* ^2^H values from 2005, 2006 and 2010 were more positive compared to 2007, 2008 and 2009 with a difference of ∼34 *‰* between the most depleted (2009) and most enriched (2005) years, respectively. Similarly, feather *δ* ^13^C suggested an isotopic enrichment of approximately 1 *‰* in 2008 and 2010 relative to 2007 and 2009 (Table 1; Figure 1B). There was greater overlap in the inter-annual variation in *δ* ^15^N, with feathers grown in 2007 being 1.4 to 0.7 *‰* more enriched than those grown in 2008 an 2009, respectively (Table 1; Figure 1C).

**Table 1:** Comparison of the multi-isotopic composition (*δ* ^2^H, *δ* ^13^C, *δ* ^15^N) of Aftrotropical grown house martin feathers from birds breeding in The Netherlands from 2005 to 2010.

	2005^a^			2006^b^			2007^c^			2008^d^			2009^e^			2010^f^		
Variable	n	Mean	SD	N	Mean	SD	n	Mean	SD	n	Mean	SD	n	Mean	SD	n	Mean	SD
δ^2^H_f_	20	−29.1^c,d,e,f^	2	27	−29.9^c,d,e,f^	2.1	71	−48.4^a,b,d,e,f^	2.4	55	−42.0^a,b,c,e^	2.5	84	−58.4^a,b,c,d,f^	2.4	51	−40.2^a,b,c,e^	2.8
δ^13^C_f_							75	−16.5^e^	0.3	54	−16.6	0.3	84	−17.2^c^	0.3	42	−16.6	0.3
δ^15^N_f_							75	10.7	0.1	54	10.2	0.1	84	10.2	0.2	42	10.7	0.2

Significant differences from Tukey’s multiple comparison tests are from a linear mixed-effects models (see Methods) are denoted using superscript letters.

Our multi-isotope assignment algorithm, based on long-term isotopic averages, placed our adult house martin sample almost entirely into two out of the four possible isotopic clusters of Africa (Table S2). However, our simulation analysis revealed that the overwhelming majority of individuals (154±2) were placed in Cluster 2, with only 15 (±2) samples assigned to Cluster 1 and two (±1) samples consistent with Cluster 3. None of the sampled house martins were assigned to Cluster 4. There was slight variance in the distribution of assignments between isotopic clusters across simulations and criteria for placing individuals to clusters based on frequency assignment across simulations (Table S2); however, the overall strong association with Cluster 2 remained.

**Figure 2:**
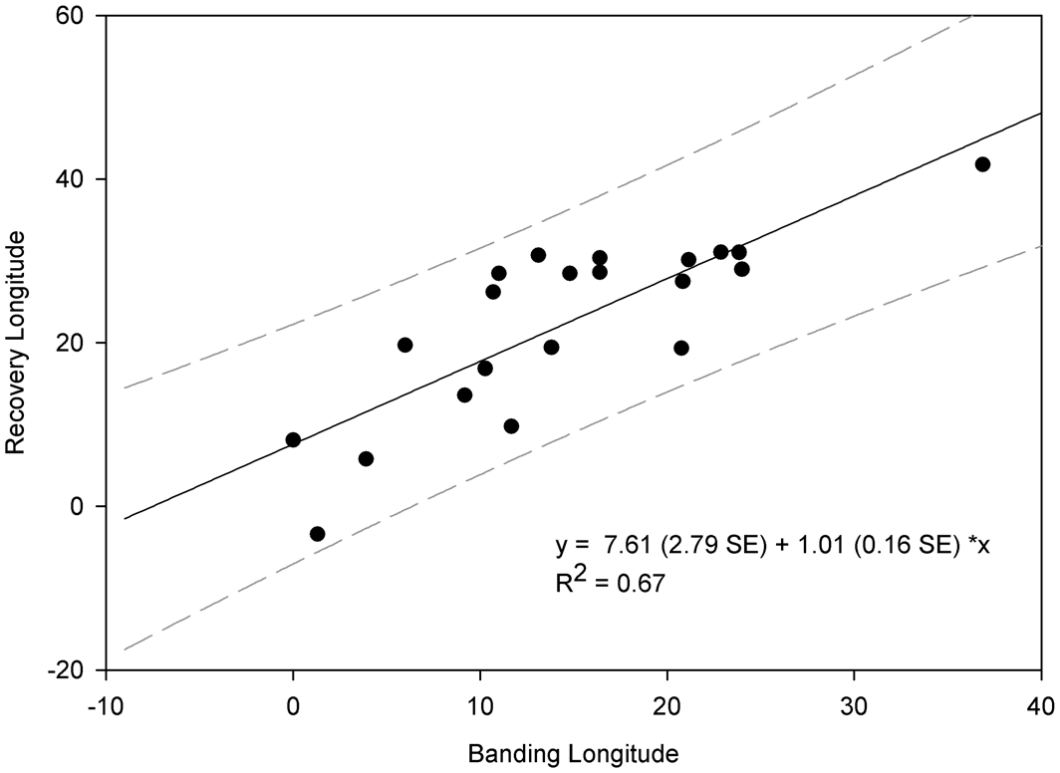
Relationship between longitude of ringing versus longitude of recovery for 21 trans-Sahara house martin ring-recoveries. Ordinary least squares regression is shown (solid line) with 95% prediction intervals (gray dashes).

**Figure 3:**
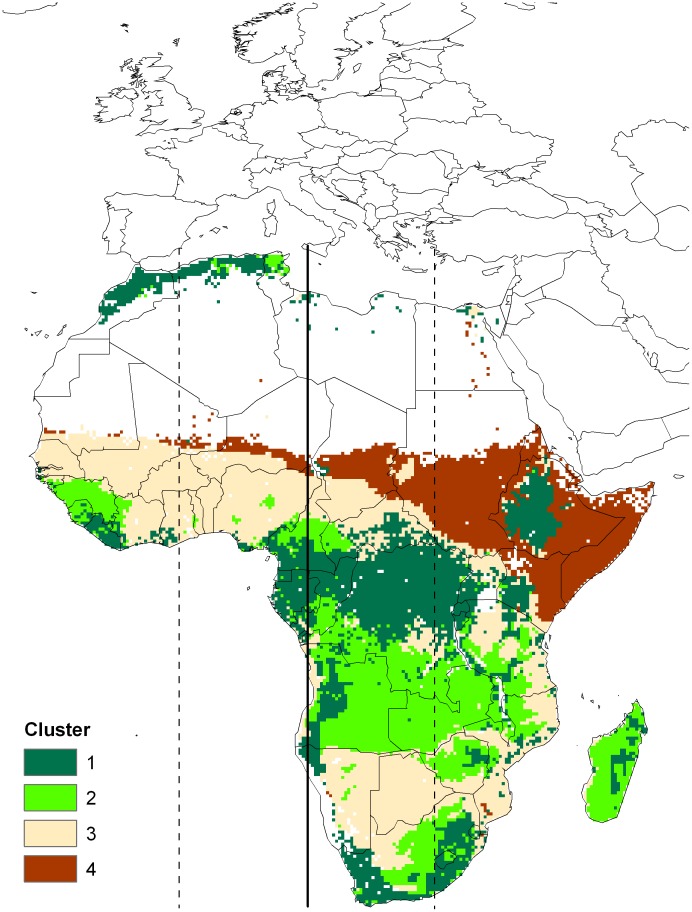
Isotopic clusters of Africa to which the house martin sample data were assigned. Isotopic clusters are from [10]. Solid line depicts predicted mean wintering longitude for birds sampled in Gaast, The Netherlands and dashed lines indicate 95% prediction interval around the mean. Predicted wintering longitude and 95% prediction intervals were derived from data in Figure 2.

House martins are classified as wintering across Africa south of 20°N [23]. Analysis of ring recovery data however, suggests a link between longitude of occupancy on the breeding and wintering grounds (Figure 2). Based upon this relationship between longitude of recovery and longitude of ringing, house martins breeding at Gaast are predicted to winter in a region centered on central Cameroon (13° longitude with a 95% prediction interval of 1°E - 27° W). This region centred on the 13° longitude is depicted in Figure 3 that also shows the locations of the unique feather isotopic clusters predicted for Africa. This exercise emphasized those regions of Cluster 2 most likely to be the regions where our Dutch-breeding house martins molted in winter. Thus, birds were assigned to wintering areas consistent with Cameroon, the Congo Basin, and fragments of Togo and Nigeria to the north and primarily Angola and Zambia to the south.

## Discussion

Using a triple isotope (*δ* ^2^H, *δ* ^13^C, *δ* ^15^N) feather basemap for Africa which portrayed four isotopically distinct clusters and ring recovery data that pointed toward a longitudinal band centred on Cameroon, we assigned the wintering origins of our adult house martins from the Netherlands primarily to regions in West Africa, especially central Cameroon and the southern Congo. While our approach relied on weak ring recovery data to provide constraints on longitude of wintering grounds, our identification of these areas of West Africa agreed remarkably well with non-isotopic work by [6] and [24]. Those researchers predicted the wintering grounds of house martin based on the identification of areas with the best covariation between annual population indices [6] and annual survival [24] of house martins on the breeding grounds in western Europe and the Normalized Differential Vegetation Index (NDVI; a satellite derived measure of “green-up” related to rainfall), in Africa. That approach was based on the assumption that years with higher rainfall and subsequent green-up in Africa would be reflected in better overwinter survival of house martins returning from those areas to Europe. Impressively, [6] in their investigation of putative origins for house martins breeding in northern Italy described wintering regions in Sierra Leone, Ivory Coast and Guinea all in our feather isotope Cluster 2 as well as regions further east in Cameroon and the northern Congo (see http://www.ace-eco.org/vol6/iss1/art3/figure2.html). This, together with the analysis by [24] for hirundines in Britain, suggest that our findings for house martins breeding at a single site in The Netherlands is likely to be true for house martins breeding in western Europe. Such distinct migratory connectivity has immense implications for conservation and emphasizes the value of these regions as wintering habitat to aerial insectivores, and likely other migrant species breeding in Europe.

We acknowledge that the isotopic clusters for Africa used to assign origins of overwintering house martins present some ambiguity due to the lack of continuous isotopic gradients. Thus, while we strongly suspect that the Dutch house martins overwintered near Cameroon and the Congo, we cannot rule out the use of a band from Angola across Zambia and, as suggested by [6], the far west forested regions of Sierra Leone, Guinea and Ivory Coast. Indeed, using only feather *δ* ^2^H data, Angola and Zambia were indicated as part of the solution space of origins of our Dutch-breeding martins in four of the six years of our investigation (Figure S1, Text S1). Currently, year-specific feather *δ* ^2^H isoscapes are not reliable for Africa due to poor coverage of the GNIP database, but strong climatic drivers such as the El-Nino Southern Oscillation and Indian Dipole Mode [25] have been linked to the isotopic composition of rainfall in Africa [26], likely driving inter-annual variation in the isoscapes as suggested by inter-annual variation in feather *δ* ^2^H over our six-year dataset. By using a multi-isotope approach based on long-term climatic drivers and a relatively long-term investigation of birds from a single colony, our interpretations are more robust than those based on a single isotope or single year of investigation. Moreover, by combining inferences from other tools such as ring return data and population parameters associated with long-term patterns of the NDVI [6], [24], the weight of evidence strongly suggests a common result, pointing toward the importance of biomes associated with western Africa with emphasis on central Cameroon and the southern Congo.

We also acknowledge that assignment of birds to winter origin using stable isotope methods may bias our perceptions as these data only provide information on molt origins. Birds may move extensively before, during, and after molt on the wintering grounds. Nonetheless, molting is an important life history stage where birds may be vulnerable and requires productive local foodwebs to fuel feather growth. Thus, molt origins are likely to be of great conservation interest.

Our investigation has provided the basis of a falsifiable hypothesis that can greatly inform future research efforts. The application of light-level geolocators to track the migratory movements of individuals [27] could provide one source of independent confirmation/refutation of our result. In our case, we are now interested in using the multi-isotope approach to delineate wintering regions of Africa used by house martins breeding in eastern Europe and which likely migrate through eastern Africa.

## Supporting Information

Figure S1 Likelihood based assignment of house martins sampled at Gaast, The Netherlands to the *δ* ^2^H isoscape of [3] based on *δ* ^2^H analysis of feathers grown in Africa in 2005 (n = 21), 2006 (n = 27), 2007 (n = 71), 2008 (n = 66), 2009 (n = 84), and 2010 (n = 51). Red ellipses represent the approximate location of putative origins for house martins breeding in Italy based on correlation between winter Normalized Difference Vegetation Index and breeding ground population indices as reported by [16].
(TIF)

Table S1 Summary of the isotopic composition of cells within *δ* ^2^H_f_, *δ* ^13^C, and *δ* ^15^N isoscapes falling within regions defined by cluster analysis of those isoscapes. *δ* ^2^H_f_ represents a calibration of the predicted amount weighted growing season average isotopic composition of rainfall.
(DOC)

Table S2 Stable isotope composition of feathers and frequency of times the individuals was assigned to the stated isotopic province of feather growth out of 1000 simulations (see Methods) for house martins sampled in Gaast, The Netherlands.
(DOC)

Text S1 Derivation of a 3-isotope *(δ^2^H, δ^13^C, δ^15^N)* African feather isoscape.
(DOC)

## References

[1] Hagemeijer WJM, Blair MJ (1997) The EBCC atlas of European breeding birds: their distribution and abundance. London: Poyser. 903 p.

[2] Zink G (1985) Der Zug europäischer Singvögel: ein Atlas der Wiederfunde beringter Vögel 2. Schloss Möggingen, Germany: Vogelwarte Radolfzell an Max-Plank Institute fur Vergaltenphysiologue.

[3] Wernham C, Siriwardena GM, Toms M, Marchant JM, Clark JA, et al. (2002) The migration atlas: movements of the birds of Britain and Ireland. London: Poyser. 900 p.

[4] Moreau RE (1972) The Palearctic-African bird migration systems. London: Academic Press. 384 p.

[5] Linnaeus C (1757) Migrationes avium. Uppsala: Uppsala University.

[6] Ambrosini R, Orioli V, Massimino D, Bani L (2011) Identification of putative wintering areas and ecological determinants of common house-martin (*Delichon urbicum*) and common swift (*Apus apus*) breeding in northern Italy. Avian Conservation and Ecology 6: 3. http://dx.doi.org/10.5751/ACE-00439-060103. Accessed 2012 Aug 29.

[7] Holmgren N, Hedenström A (1995) The scheduling of moult in migratory birds. Evolutionary Ecology 9: 354–358.

[8] Barta Z, McNamara JM, Houston AI, Weber TP, Hedenström A, et al (2008) Optimal moult strategies in migratory birds. Philosophical Transactions of the Royal Society B 363: 211–229. 10.1098/rstb.2007.2136 PMC2606747 17681914

[9] Cramp S (1988) Handbook of the Birds of Europe, the Middle East and North Africa: The birds of the Western Palearctic; Cramp S, editor. Oxford: Oxford University Press. 1063 p.

[10] Hobson KA, Van Wilgenburg SL, Wassenaar LI, Powell RL, Still CJ, et al (2012) A multi-isotope (*δ* ^13^C, *δ* ^15^N, *δ* ^2^H) feather isoscape to assign Afrotropical migrant birds to origins. Ecosphere: in press.

[11] Piersma T, van der Velde M (2009) Breeding season-specific sex diagnostics in the monomorphic house martin *Delichon urbicum* . Bird Study 56: 127–131.

[12] Wassenaar LI, Hobson KA (2003) Comparative equilibration and online technique for determination of non-exchangeable hydrogen of keratins for animal migration studies. Isotopes in Environmental and Health Studies 39: 1–7. 10.1080/1025601031000096781 14521282

[13] Zuur AF, Ieno EN, Walker NJ, Saveliev AA, Smith GM (2009) Mixed effects models and extensions in ecology with R. New York: Springer. 574 p.

[14] Hothorn T, Bretz F, Westfall P (2008) Simultaneous inference in general parametric models. Biometrical Journal 50: 346–363. 18481363 10.1002/bimj.200810425

[15] Still CJ, Powell RL (2010) Continental-scale distributions of vegetation stable carbon isotope ratios. In: West JB, Bowen GJ, Dawson TE, Tu KP, editors. Isoscapes: understanding movements, pattern and process on Earth through isotope mapping. New York: Springer. 179–194.

[16] Craine JM, Elmore AJ, Aidar MPM, Bustamante M, Dawson TE, et al (2009) Global patterns of foliar nitrogen isotopes and their relationships with climate, mycorrhizal fungi, foliar nutrient concentrations, and nitrogen availability. New Phytologist 183: 980–992. 19563444 10.1111/j.1469-8137.2009.02917.x

[17] Bowen GJ, Wassenaar LI, Hobson KA (2005) Global application of stable hydrogen and oxygen isotopes to wildlife forensics. Oecologia 143: 337–348. 15726429 10.1007/s00442-004-1813-y

[18] Royle JA, Rubenstein DR (2004) The role of species abundance in determining breeding origins of migratory birds with stable isotopes. Ecological Applications 14: 1780–1788.

[19] R Development Core Team (2011) R: A language and environment for statistical computing. Vienna, Austria: R Foundation for Statistical Computing. ISBN 3–900051–07–0.

[20] Genz A, Bretz F (2009) Computation of multivariate normal and t probabilities. Lecture notes in statistics. Heidelberg: Springer-Verlage.

[21] Genz A, Bretz F, Miwa T, Mi X, Leisch F, et al (2011) mvtnorm: multivariate normal and t distributions. R package version 0.9–99. http://CRAN.R-project.org/package=mvtnorm ed. Accessed 2012 Aug 29.

[22] Hill LA (1997) Trans-Sahara recoveries of house martins *Delichon urbica*, with discussion on ringing, roosting and sightings in Africa. Safring News 26: 7–12.

[23] Turner A (2004) Family Hirundinidae (Swallows and Martins). In: Hoyo Jd, Elliot A, Christie DA, editors. Handbook of the birds of the world. Barcelona, Spain: Lynx Edicions. 602–638.

[24] Robinson RA, Balmer DE, Marchant JM (2008) Survival rates of hirundines in relation to British and African rainfall. Ringing & Migration 24: 1–6.

[25] Williams CA, Hanan NP (2011) ENSO and IOD teleconnections for African ecosystems: evidence of destructive interference between climate oscillations. Biogeosciences 8: 27–40.

[26] Vuille M, Werner M, Bradley RS, Chan R, Keimig F (2005) Stable isotopes in East African precipitation record Indian Ocean zonal mode. Geophysical Research Letters 32: L21705.

[27] Stutchbury BJM, Tarof SA, Done T, Gow E, Kramer PM, et al (2009) Tracking long-distance songbird migration by using geolocators. Science 323: 896. 19213909 10.1126/science.1166664

