# Supplementary figures and images for: Solving a Migration Riddle Using Isoscapes: House Martins from a Dutch Village Winter over West Africa
Source: PLoS One. 2012 Sep 21;7(9):e45005. doi: 10.1371/journal.pone.0045005 (PMC3448620; doi:10.1371/journal.pone.0045005)

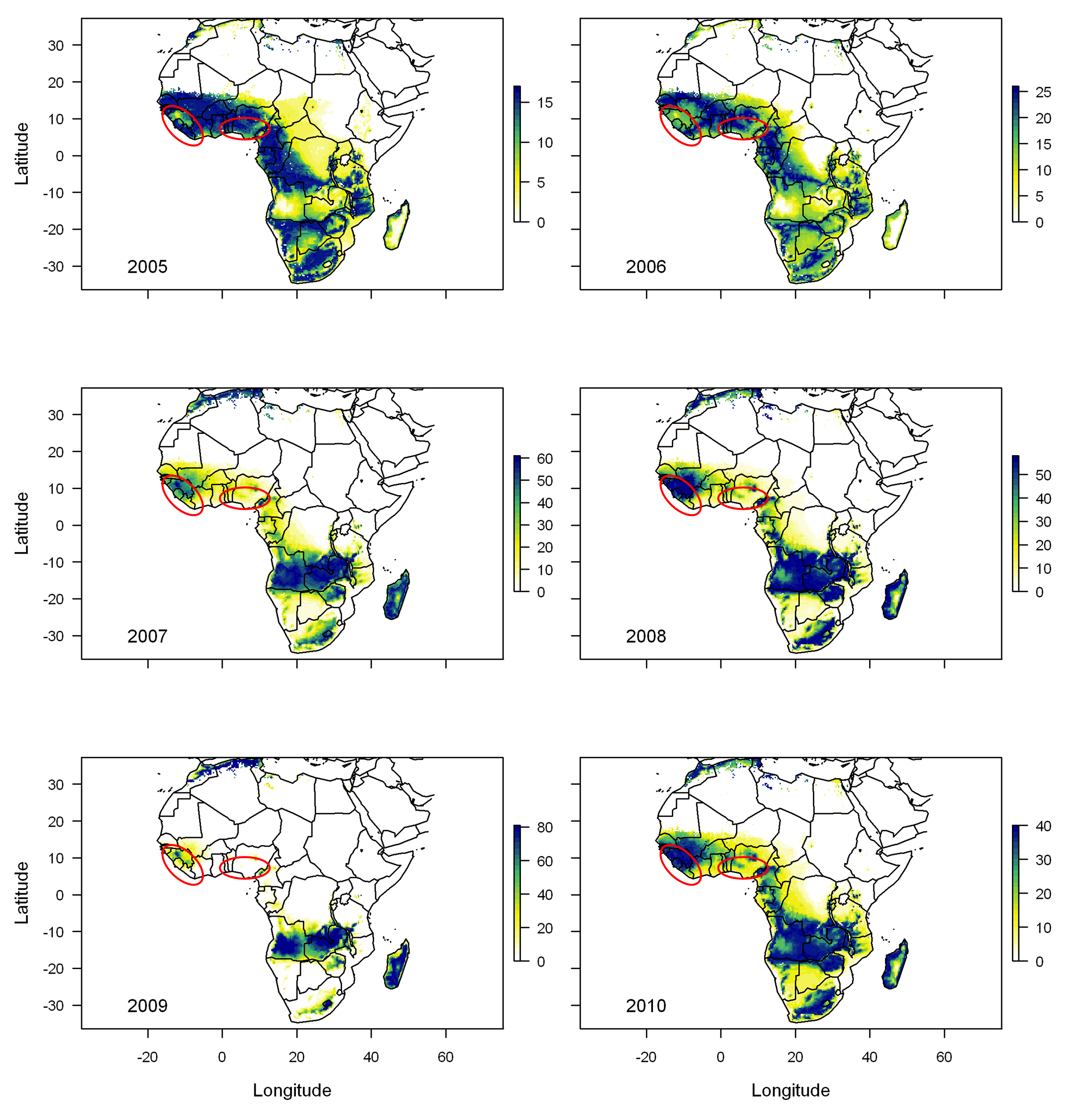

Supplement: Figure S1 — Likelihood based assignment of house martins sampled at Gaast, The Netherlands to the δ 2H isoscape of [3] based on δ 2H analysis of feathers grown in Africa in 2005 (n = 21), 2006 (n = 27), 2007 (n = 71), 2008 (n = 66), 2009 (n = 84), and 2010 (n = 51). Red ellipses represent the approximate location of putative origins for house martins breeding in Italy based on correlation between winter Normalized Difference Vegetation Index and breeding ground population indices as reported by [16]. (TIF) [file pone.0045005.s001.tif]
