# Supplementary material for: Solving a Migration Riddle Using Isoscapes: House Martins from a Dutch Village Winter over West Africa
Source: PLoS One. 2012 Sep 21;7(9):e45005. doi: 10.1371/journal.pone.0045005 (PMC3448620; doi:10.1371/journal.pone.0045005)
Supplement: Table S1 — Summary of the isotopic composition of cells within δ 2Hf, δ 13C, and δ 15N isoscapes falling within regions defined by cluster analysis of those isoscapes. δ 2Hf represents a calibration of the predicted amount weighted growing season average isotopic composition of rainfall. (DOC) [file pone.0045005.s002.doc]

|  | *δ*^2^H | | | | *δ*^13^C | | | | *δ*^15^N | | | |
| --- | --- | --- | --- | --- | --- | --- | --- | --- | --- | --- | --- | --- |
| Cluster | Mean | SD | Min | Max | Mean | SD | Min | Max | Mean | SD | Min | Max |
| 1 | -24.3 | 14.9 | -68.7 | 14.5 | -22.1 | 2.5 | -25.0 | -13.6 | 9.4 | 0.8 | 5.4 | 13.7 |
| 2 | -37.5 | 8.4 | -76.3 | -7.7 | -14.4 | 2.3 | -25.0 | -10.0 | 9.5 | 0.5 | 5.7 | 10.8 |
| 3 | -22.2 | 7.5 | -39.5 | 6.0 | -12.3 | 2.0 | -25.0 | -10.0 | 10.8 | 0.8 | 8.5 | 14.1 |
| 4 | 6.7 | 12.7 | -25.7 | 35.8 | -11.1 | 1.9 | -25.0 | -10.0 | 11.8 | 0.9 | 9.1 | 20.4 |
