# Supplementary material for: Solving a Migration Riddle Using Isoscapes: House Martins from a Dutch Village Winter over West Africa
Source: PLoS One. 2012 Sep 21;7(9):e45005. doi: 10.1371/journal.pone.0045005 (PMC3448620; doi:10.1371/journal.pone.0045005)
Supplement: Table S2 — Stable isotope composition of feathers and frequency of times the individuals was assigned to the stated isotopic province of feather growth out of 1000 simulations (see Methods) for house martins sampled in Gaast, The Netherlands. (DOC) [file pone.0045005.s003.doc]

|  |  |  |  | Isotopic Province | | | |
| --- | --- | --- | --- | --- | --- | --- | --- |
| Ring number | *δ*^2^H_f_ | *δ*^13^C | *δ*^15^N | 1 | 2 | 3 | 4 |
| 9803589 | -69.4 | -15.6 | 9.9 | 0.00 | 1.00 | 0.00 | 0.00 |
| AL98102 | -38.8 | -15.6 | 11.6 | 0.00 | 0.97 | 0.03 | 0.00 |
| AL98108 | -28.9 | -16.9 | 10.9 | 0.00 | 1.00 | 0.00 | 0.00 |
| AL98111 | -32.8 | -15.0 | 11.1 | 0.00 | 0.89 | 0.11 | 0.00 |
| AL98116 | -32.2 | -14.6 | 11.3 | 0.00 | 0.65 | 0.35 | 0.00 |
| AL98122 | -26.1 | -15.5 | 11.0 | 0.00 | 0.97 | 0.03 | 0.00 |
| AL98133 | -31.4 | -16.3 | 11.1 | 0.00 | 1.00 | 0.00 | 0.00 |
| AL98137 | -36.1 | -15.4 | 10.7 | 0.00 | 1.00 | 0.00 | 0.00 |
| AL98146 | -31.5 | -18.3 | 10.0 | 0.43 | 0.57 | 0.00 | 0.00 |
| AL98149 | -23.5 | -16.2 | 10.6 | 0.00 | 1.00 | 0.00 | 0.00 |
| AL98150 | -49.8 | -17.4 | 11.0 | 0.00 | 1.00 | 0.00 | 0.00 |
| AL98151 | -20.3 | -17.0 | 9.8 | 0.00 | 1.00 | 0.00 | 0.00 |
| AL98152 | -62.6 | -17.2 | 11.5 | 0.00 | 1.00 | 0.00 | 0.00 |
| AL98153 | -57.6 | -17.4 | 10.3 | 0.00 | 1.00 | 0.00 | 0.00 |
| AL98154 | -58.4 | -18.7 | 11.1 | 0.16 | 0.84 | 0.00 | 0.00 |
| AL98155 | -57.2 | -16.7 | 11.0 | 0.00 | 1.00 | 0.00 | 0.00 |
| AL98156 | -58.5 | -18.7 | 11.0 | 0.16 | 0.84 | 0.00 | 0.00 |
| AL98157 | -53.6 | -18.3 | 9.5 | 0.15 | 0.86 | 0.00 | 0.00 |
| AL98158 | -59.3 | -15.3 | 11.5 | 0.00 | 1.00 | 0.00 | 0.00 |
| AL98159 | -60.6 | -17.3 | 11.1 | 0.00 | 1.00 | 0.00 | 0.00 |
| AL98160 | -44.8 | -15.9 | 10.5 | 0.00 | 1.00 | 0.00 | 0.00 |
| AL98161 | -61.6 | -15.3 | 11.6 | 0.00 | 1.00 | 0.00 | 0.00 |
| AL98162 | -53.9 | -15.0 | 11.5 | 0.00 | 0.98 | 0.02 | 0.00 |
| AL98163 | -54.4 | -17.4 | 10.2 | 0.00 | 1.00 | 0.00 | 0.00 |
| AL98165 | -70.1 | -16.9 | 10.4 | 0.00 | 1.00 | 0.00 | 0.00 |
| AL98167 | -42.1 | -18.7 | 10.0 | 0.60 | 0.40 | 0.00 | 0.00 |
| AL98168 | -62.9 | -17.5 | 11.0 | 0.00 | 1.00 | 0.00 | 0.00 |
| AL98169 | -58.4 | -17.8 | 10.3 | 0.00 | 1.00 | 0.00 | 0.00 |
| AL98176 | -63.5 | -16.5 | 11.0 | 0.00 | 1.00 | 0.00 | 0.00 |
| AL98178 | -62.5 | -15.8 | 13.1 | 0.00 | 0.96 | 0.04 | 0.00 |
| AL98179 | -61.2 | -14.4 | 11.1 | 0.00 | 0.99 | 0.01 | 0.00 |
| AL98183 | -62.5 | -16.7 | 10.5 | 0.00 | 1.00 | 0.00 | 0.00 |
| AL98184 | -54.0 | -17.4 | 10.3 | 0.00 | 1.00 | 0.00 | 0.00 |
| AL98187 | -53.1 | -17.3 | 10.8 | 0.00 | 1.00 | 0.00 | 0.00 |
| AL98188 | -60.7 | -15.9 | 10.6 | 0.00 | 1.00 | 0.00 | 0.00 |
| AL98189 | -59.4 | -15.6 | 11.4 | 0.00 | 1.00 | 0.00 | 0.00 |
| AL98193 | -59.6 | -15.6 | 11.4 | 0.00 | 1.00 | 0.00 | 0.00 |
| AL98194 | -45.1 | -16.9 | 9.8 | 0.00 | 1.00 | 0.00 | 0.00 |
| AL98195 | -48.6 | -14.5 | 9.9 | 0.00 | 1.00 | 0.00 | 0.00 |
| AL98196 | -51.6 | -15.8 | 10.8 | 0.00 | 1.00 | 0.00 | 0.00 |
| AL98197 | -44.0 | -15.0 | 10.5 | 0.00 | 1.00 | 0.00 | 0.00 |
| AL98198 | -54.9 | -16.0 | 11.6 | 0.00 | 1.00 | 0.00 | 0.00 |
| AL98199 | -62.8 | -15.5 | 12.8 | 0.00 | 0.94 | 0.06 | 0.00 |
| AL98200 | -65.9 | -14.8 | 9.6 | 0.00 | 1.00 | 0.00 | 0.00 |
| AL98201 | -40.0 | -15.4 | 10.6 | 0.00 | 1.00 | 0.00 | 0.00 |
| AL98202 | -54.2 | -17.2 | 9.9 | 0.00 | 1.00 | 0.00 | 0.00 |
| AL98207 | -46.8 | -16.5 | 11.2 | 0.00 | 1.00 | 0.00 | 0.00 |
| AL98208 | -55.3 | -15.9 | 10.5 | 0.00 | 1.00 | 0.00 | 0.00 |
| AL98209 | -46.5 | -16.1 | 10.1 | 0.00 | 1.00 | 0.00 | 0.00 |
| AL98210 | -44.4 | -17.7 | 9.5 | 0.02 | 0.99 | 0.00 | 0.00 |
| AL98211 | -40.1 | -19.3 | 10.3 | 0.99 | 0.01 | 0.00 | 0.00 |
| AL98212 | -37.5 | -15.9 | 9.8 | 0.00 | 1.00 | 0.00 | 0.00 |
| AL98213 | -32.6 | -16.0 | 10.0 | 0.00 | 1.00 | 0.00 | 0.00 |
| AL98214 | -48.7 | -16.3 | 11.2 | 0.00 | 1.00 | 0.00 | 0.00 |
| AL98215 | -46.2 | -15.7 | 11.1 | 0.00 | 1.00 | 0.00 | 0.00 |
| AL98217 | -42.5 | -14.3 | 10.1 | 0.00 | 1.00 | 0.00 | 0.00 |
| AL98218 | -48.1 | -17.9 | 10.0 | 0.02 | 0.98 | 0.00 | 0.00 |
| AL98220 | -51.5 | -17.2 | 11.0 | 0.00 | 1.00 | 0.00 | 0.00 |
| AL98221 | -45.0 | -15.8 | 11.0 | 0.00 | 1.00 | 0.00 | 0.00 |
| AL98222 | -52.1 | -16.7 | 11.0 | 0.00 | 1.00 | 0.00 | 0.00 |
| AL98223 | -47.1 | -16.9 | 10.1 | 0.00 | 1.00 | 0.00 | 0.00 |
| AL98227 | -48.9 | -18.3 | 10.3 | 0.12 | 0.88 | 0.00 | 0.00 |
| AL98228 | -37.2 | -16.6 | 9.5 | 0.00 | 1.00 | 0.00 | 0.00 |
| AL98229 | -42.1 | -16.1 | 9.5 | 0.00 | 1.00 | 0.00 | 0.00 |
| AL98230 | -53.1 | -17.6 | 11.0 | 0.00 | 1.00 | 0.00 | 0.00 |
| AL98231 | -45.9 | -17.4 | 11.1 | 0.00 | 1.00 | 0.00 | 0.00 |
| AL98232 | -53.5 | -15.5 | 11.0 | 0.00 | 1.00 | 0.00 | 0.00 |
| AL98233 | -41.3 | -16.9 | 10.1 | 0.00 | 1.00 | 0.00 | 0.00 |
| AL98234 | -51.3 | -19.0 | 10.6 | 0.66 | 0.34 | 0.00 | 0.00 |
| AL98235 | -28.2 | -16.3 | 10.3 | 0.00 | 1.00 | 0.00 | 0.00 |
| AL98236 | -36.2 | -16.4 | 11.2 | 0.00 | 1.00 | 0.00 | 0.00 |
| AL98237 | -35.8 | -19.7 | 10.3 | 1.00 | 0.00 | 0.00 | 0.00 |
| AL98240 | -45.1 | -16.7 | 10.4 | 0.00 | 1.00 | 0.00 | 0.00 |
| AL98241 | -44.2 | -13.0 | 10.5 | 0.00 | 0.46 | 0.54 | 0.00 |
| AL98242 | -45.9 | -16.3 | 10.8 | 0.00 | 1.00 | 0.00 | 0.00 |
| AL98243 | -28.1 | -17.7 | 9.5 | 0.10 | 0.90 | 0.00 | 0.00 |
| AL98245 | -26.7 | -16.3 | 8.8 | 0.00 | 1.00 | 0.00 | 0.00 |
| AL98246 | -44.8 | -16.2 | 9.8 | 0.00 | 1.00 | 0.00 | 0.00 |
| AL98247 | -31.9 | -16.9 | 9.1 | 0.00 | 1.00 | 0.00 | 0.00 |
| AL98248 | -52.8 | -18.5 | 9.8 | 0.31 | 0.69 | 0.00 | 0.00 |
| AL98249 | -51.0 | -17.9 | 10.8 | 0.00 | 1.00 | 0.00 | 0.00 |
| AL98251 | -34.3 | -18.5 | 9.3 | 0.69 | 0.31 | 0.00 | 0.00 |
| AL98253 | -40.5 | -16.8 | 10.7 | 0.00 | 1.00 | 0.00 | 0.00 |
| AL98255 | -46.0 | -15.6 | 11.6 | 0.00 | 1.00 | 0.00 | 0.00 |
| AL98257 | -47.6 | -16.7 | 10.3 | 0.00 | 1.00 | 0.00 | 0.00 |
| AL98259 | -37.9 | -15.7 | 10.6 | 0.00 | 1.00 | 0.00 | 0.00 |
| AL98263 | -51.5 | -17.6 | 9.1 | 0.01 | 0.99 | 0.00 | 0.00 |
| AL98264 | -33.2 | -17.2 | 9.9 | 0.00 | 1.00 | 0.00 | 0.00 |
| AL98265 | -33.6 | -18.4 | 9.5 | 0.60 | 0.40 | 0.00 | 0.00 |
| AL98267 | -28.2 | -16.7 | 8.8 | 0.00 | 1.00 | 0.00 | 0.00 |
| AL98274 | -40.6 | -16.9 | 8.8 | 0.00 | 1.00 | 0.00 | 0.00 |
| AL98275 | -48.5 | -15.9 | 10.9 | 0.00 | 1.00 | 0.00 | 0.00 |
| AL98276 | -48.3 | -15.7 | 10.8 | 0.00 | 1.00 | 0.00 | 0.00 |
| AL98277 | -55.3 | -16.6 | 10.6 | 0.00 | 1.00 | 0.00 | 0.00 |
| AL98278 | -42.7 | -19.7 | 12.1 | 0.98 | 0.02 | 0.00 | 0.00 |
| AL98279 | -42.8 | -15.6 | 11.0 | 0.00 | 1.00 | 0.00 | 0.00 |
| AL98283 | -45.4 | -18.1 | 9.6 | 0.11 | 0.89 | 0.00 | 0.00 |
| AL98289 | -53.2 | -14.2 | 12.1 | 0.00 | 0.50 | 0.50 | 0.00 |
| AL98290 | -4.0 | -14.3 | 9.9 | 0.00 | 0.63 | 0.35 | 0.02 |
| AL98293 | -56.9 | -14.9 | 10.9 | 0.00 | 1.00 | 0.00 | 0.00 |
| AP10002 | -58.9 | -16.0 | 11.2 | 0.00 | 1.00 | 0.00 | 0.00 |
| AP10014 | -60.3 | -14.7 | 10.8 | 0.00 | 1.00 | 0.00 | 0.00 |
| AP10023 | -61.1 | -14.3 | 11.9 | 0.00 | 0.84 | 0.16 | 0.00 |
| AP10030 | -62.1 | -17.7 | 10.3 | 0.00 | 1.00 | 0.00 | 0.00 |
| AP10033 | -70.3 | -18.1 | 10.1 | 0.01 | 0.99 | 0.00 | 0.00 |
| AP10038 | -55.5 | -15.8 | 9.9 | 0.00 | 1.00 | 0.00 | 0.00 |
| AP10039 | -52.9 | -19.5 | 9.7 | 0.98 | 0.02 | 0.00 | 0.00 |
| AP10040 | -57.2 | -15.5 | 8.9 | 0.00 | 1.00 | 0.00 | 0.00 |
| AP10041 | -61.1 | -16.9 | 9.8 | 0.00 | 1.00 | 0.00 | 0.00 |
| AP10042 | -43.7 | -16.7 | 9.3 | 0.00 | 1.00 | 0.00 | 0.00 |
| AP10044 | -54.9 | -15.4 | 9.3 | 0.00 | 1.00 | 0.00 | 0.00 |
| AP10046 | -49.5 | -18.0 | 10.2 | 0.03 | 0.97 | 0.00 | 0.00 |
| AP10047 | -67.4 | -16.9 | 10.6 | 0.00 | 1.00 | 0.00 | 0.00 |
| AP10048 | -50.8 | -17.3 | 9.9 | 0.00 | 1.00 | 0.00 | 0.00 |
| AP10049 | -50.5 | -16.7 | 8.0 | 0.00 | 1.00 | 0.00 | 0.00 |
| AP10050 | -62.2 | -17.9 | 10.3 | 0.00 | 1.00 | 0.00 | 0.00 |
| AP10101 | -43.9 | -16.6 | 9.6 | 0.00 | 1.00 | 0.00 | 0.00 |
| AP10102 | -62.2 | -16.5 | 10.0 | 0.00 | 1.00 | 0.00 | 0.00 |
| AP10103 | -63.0 | -16.7 | 9.5 | 0.00 | 1.00 | 0.00 | 0.00 |
| AP10104 | -59.4 | -16.1 | 8.9 | 0.00 | 1.00 | 0.00 | 0.00 |
| AP10105 | -60.8 | -18.1 | 8.7 | 0.06 | 0.94 | 0.00 | 0.00 |
| AP10106 | -63.0 | -17.0 | 8.8 | 0.00 | 1.00 | 0.00 | 0.00 |
| AP10107 | -50.9 | -13.9 | 9.2 | 0.00 | 1.00 | 0.00 | 0.00 |
| AP10108 | -56.7 | -17.6 | 9.5 | 0.00 | 1.00 | 0.00 | 0.00 |
| AP10109 | -54.1 | -16.1 | 10.3 | 0.00 | 1.00 | 0.00 | 0.00 |
| AP10111 | -48.9 | -17.7 | 9.2 | 0.01 | 0.99 | 0.00 | 0.00 |
| AP10112 | -63.2 | -18.8 | 10.2 | 0.32 | 0.68 | 0.00 | 0.00 |
| AP10114 | -72.4 | -15.8 | 9.7 | 0.00 | 1.00 | 0.00 | 0.00 |
| AP10116 | -62.4 | -15.9 | 9.1 | 0.00 | 1.00 | 0.00 | 0.00 |
| AP10118 | -39.1 | -17.1 | 9.3 | 0.00 | 1.00 | 0.00 | 0.00 |
| AP10119 | -57.4 | -15.2 | 9.4 | 0.00 | 1.00 | 0.00 | 0.00 |
| AP10120 | -52.8 | -17.2 | 9.2 | 0.00 | 1.00 | 0.00 | 0.00 |
| AP10121 | -69.4 | -17.4 | 11.2 | 0.00 | 1.00 | 0.00 | 0.00 |
| AP10122 | -75.4 | -17.8 | 9.6 | 0.00 | 1.00 | 0.00 | 0.00 |
| AP10124 | -58.9 | -21.1 | 15.9 | 1.00 | 0.00 | 0.00 | 0.00 |
| AP10125 | -51.0 | -17.2 | 8.5 | 0.00 | 1.00 | 0.00 | 0.00 |
| AP10127 | -43.1 | -16.1 | 9.3 | 0.00 | 1.00 | 0.00 | 0.00 |
| AP10128 | -55.4 | -15.9 | 9.3 | 0.00 | 1.00 | 0.00 | 0.00 |
| AP10129 | -46.2 | -15.2 | 10.5 | 0.00 | 1.00 | 0.00 | 0.00 |
| AP10130 | -64.9 | -16.6 | 10.3 | 0.00 | 1.00 | 0.00 | 0.00 |
| AP10135 | -57.2 | -16.6 | 11.3 | 0.00 | 1.00 | 0.00 | 0.00 |
| AP10138 | -50.0 | -17.9 | 9.5 | 0.02 | 0.98 | 0.00 | 0.00 |
| AP10139 | -67.2 | -16.1 | 10.7 | 0.00 | 1.00 | 0.00 | 0.00 |
| AP10140 | -56.6 | -16.9 | 10.0 | 0.00 | 1.00 | 0.00 | 0.00 |
| AP10142 | -54.5 | -16.4 | 9.9 | 0.00 | 1.00 | 0.00 | 0.00 |
| AP10144 | -54.1 | -18.5 | 9.5 | 0.31 | 0.69 | 0.00 | 0.00 |
| AP10145 | -59.0 | -19.0 | 9.6 | 0.74 | 0.26 | 0.00 | 0.00 |
| AP10146 | -106.9 | -18.1 | 8.9 | 0.00 | 1.00 | 0.00 | 0.00 |
| AP10147 | -37.4 | -17.3 | 9.5 | 0.00 | 1.00 | 0.00 | 0.00 |
| AP10148 | -65.5 | -16.8 | 9.4 | 0.00 | 1.00 | 0.00 | 0.00 |
| AP10151 | -73.8 | -19.8 | 16.7 | 0.07 | 0.93 | 0.00 | 0.00 |
| AP10156 | -54.8 | -17.4 | 13.2 | 0.00 | 1.00 | 0.00 | 0.00 |
| AP10157 | -53.6 | -16.3 | 8.7 | 0.00 | 1.00 | 0.00 | 0.00 |
| AP10158 | -56.5 | -17.4 | 9.8 | 0.00 | 1.00 | 0.00 | 0.00 |
| AP10159 | -69.0 | -19.8 | 9.3 | 0.99 | 0.01 | 0.00 | 0.00 |
| AP10160 | -65.0 | -16.1 | 9.5 | 0.00 | 1.00 | 0.00 | 0.00 |
| AP10161 | -64.7 | -18.8 | 9.8 | 0.34 | 0.66 | 0.00 | 0.00 |
| AP10163 | -57.8 | -21.4 | 11.4 | 1.00 | 0.00 | 0.00 | 0.00 |
| AP10164 | -47.0 | -16.2 | 10.1 | 0.00 | 1.00 | 0.00 | 0.00 |
| AP10165 | -81.4 | -21.1 | 16.5 | 0.97 | 0.04 | 0.00 | 0.00 |
| AP10166 | -45.5 | -16.1 | 9.3 | 0.00 | 1.00 | 0.00 | 0.00 |
| AP10168 | -42.9 | -17.0 | 9.0 | 0.00 | 1.00 | 0.00 | 0.00 |
| AP10174 | -52.3 | -17.2 | 11.3 | 0.00 | 1.00 | 0.00 | 0.00 |
| AP10175 | -52.5 | -20.3 | 16.5 | 0.85 | 0.15 | 0.00 | 0.00 |
| AP10178 | -56.4 | -17.5 | 9.8 | 0.00 | 1.00 | 0.00 | 0.00 |
| AP10179 | -48.5 | -16.0 | 10.1 | 0.00 | 1.00 | 0.00 | 0.00 |
| AP10181 | -46.7 | -15.3 | 10.1 | 0.00 | 1.00 | 0.00 | 0.00 |
| AP10183 | -54.9 | -17.1 | 11.4 | 0.00 | 1.00 | 0.00 | 0.00 |
| AP10184 | -65.0 | -15.1 | 10.7 | 0.00 | 1.00 | 0.00 | 0.00 |
| AP10185 | -57.5 | -16.6 | 10.1 | 0.00 | 1.00 | 0.00 | 0.00 |
| un-ringed | -69.4 | -15.7 | 10.0 | 0.00 | 1.00 | 0.00 | 0.00 |
