# Supplementary material for: Solving a Migration Riddle Using Isoscapes: House Martins from a Dutch Village Winter over West Africa
Source: PLoS One. 2012 Sep 21;7(9):e45005. doi: 10.1371/journal.pone.0045005 (PMC3448620; doi:10.1371/journal.pone.0045005)
Supplement: Text S1 — Derivation of a 3-isotope (δ2H, δ13C, δ15N) African feather isoscape. (DOC) [file pone.0045005.s004.doc]

We obtained spatially explicit raster surfaces of the theoretical distribution of plant *δ*^13^C values from . This ^13^C raster isoscape was created by modeling the relative cover of C3 versus C4 plants, with integrated data on fractional cover of crop types to account for managed agroecosystems that may violate natural climatic constraints. Predicted *δ*^13^C value was based on applying endmember *δ*^13^C values for each photosynthetic type within the land grid cells

A plant *δ*^15^N isoscape based on a model developed by was used to derive subsequent feather *δ*^15^N isoscapes. The plant *δ*^15^N model was derived from covariation between climatic variables (mean annual precipitation, mean annual temperature) and a survey of plant foliar *δ*^15^N values worldwide . We used the amount-weighted mean annual growing season precipitation *δ*^2^H isoscape (hereafter *δ*^2^H_p_) derived by ; available at www.waterisotopes.org. The *δ*^2^H_p_ surface was based primarily upon the nearly 50-year GNIP dataset provided by the International Atomic Energy Agency (IAEA). used an algorithm that accounted for altitude and latitude, and was weighted by long-term monthly precipitation amount surfaces.

Isoscapes required final calibration into units consistent with expectations for feathers grown in Africa. We used feather *δ*^2^H values (hereafter *δ*^2^H_f_) data from known-origin songbirds reported in , and Reed warblers (P. Procházka, unpublished data), and regressed these against *δ*^2^H_p_ from the isoscape of . The resulting calibration equation (*δ*^2^H_f_ = -6.77 + 1.42 GSD), was used to recalibrate *δ*^2^H_p_ isoscape into a *δ*^2^H_f_ isoscape. To create a feather *δ*^13^C isoscape, we applied a +2 *‰* discrimination factor to the plant *δ*^13^C isoscape, assuming a +1 *‰* isotopic discrimination between plants and herbivorous insects , , and 1 *‰* discrimination between insects and bird feathers . Similarly, we applied a +5 *‰* trophic discrimination to the *δ*^15^N isoscape, assuming approximately +2.5 *‰* from plant to insect, and 2.5 *‰* from insects to feathers .

Feather isoscapes for *δ*^13^C, *δ*^15^N, and *δ*^2^H_f_ were exported from their native ArcGIS grid format to ascii raster files using ArcGIS v 9.1 (ESRI, Redlands, CA). The raster surfaces were then imported into the R statistical computing environment using the ‘raster’ package for further manipulation and analysis. The calibrated isoscapes were examined for “natural” groupings in multivariate space by cluster analysis to partition the data using the partitioning around medoids (hereafter ‘PAM’) clustering algorithm. ‘PAM’ is a form of clustering related to K-means clustering, based on medians as opposed to means , and selects data points to serve as medoids (representing potential cluster centers), and attempts to assign each data point to the nearest medoid. The algorithm sought to minimize the sum of the dissimilarities of the observations to the closest representative medoid ,. However, the algorithm is cumbersome on large data sets, thus, cluster analysis was conducted using the ‘clara’ algorithm which is based on the PAM algorithm, but selects an optimal set of medoids over a series of random samples of the data. We conducted our analyses by using Euclidean distance as the dissimilarity measure. Determining the optimal number of clusters into which the data should be divided is a major challenge in cluster analyses, thus we used a cross-validation criterion known as “prediction strength” . An initial sample of 15% of the data, was further split into prediction and validation sets in a cross-validation, and prediction strength was computed by estimating the proportion of observations in the test set in which the observation was placed to the same cluster by applying algorithms derived separately from the test and training samples . We ran iterations limiting the cluster analyses to a minimum of two and a maximum of ten clusters, with each iteration including 50 divisions of the data into training and validation sets. Data were standardized to z-scores (subtraction of the variables mean and division by the standard deviation for the given variable) prior to clustering. After selection of the optimal number of clusters, we applied the ‘clara’ algorithm to the entire data set using the specified number of cluster into which the data should be clustered. We conducted these analyses using the ‘fpc’ package within R 2.13.0 .

Analysis of data from all three (*δ*^13^C, *δ*^15^N, and *δ*^2^H) feather isoscapes using cluster analysis suggested that partitioning the data into four clusters provided the optimal number of partitions based upon theoretical prediction strength. Across 50 randomizations, sample pairs were placed in the same cluster ~88 % of the time (mean prediction strength= 0.88 ± 0.05 SD) using four clusters. Cluster 3-1 had most negative *δ*^13^C_f_ and *δ*^15^N_f_ values, and was the second- most negative cluster with respect to ^2^H_f_ (Table S1). Cluster 1 was associated with the Congo Basin, north Africa, and pockets of west Africa, southern Africa and Madagascar (Figure 3). Cluster 2 was primarily consistent with forested biomes of Cameroon, the Congo Basin, Angola, Zambia and South Africa, as well as fragments of Togo, Nigeria, Sierra Leone, Ivory Coast and Guinea north of the equator (Figure 3). Cluster 3 was associated primarily with savannah vegetation along a broad arc from Senegal south and east toward the Congo Basin, along Africa’s eastern shoreline and westward toward Namibia (Figure 3). In contrast, Cluster 4 was comparatively restricted in the horn of Africa and along portions of the north-western Sahel (Figure 3).

**Depictions of origins of house martins based only on *δ*^2^H.**

We assigned house martins to the aforementioned calibrated *δ*^2^H_f_ isoscape using a likelihood-based assignment . Thus, we used a normal probability density function to assess the likelihood that a given sample was consistent with a cell within the isoscape given *δ*^2^H_f_, the mean expected *δ*^2^H_f_ of a feather grown at that cell in the isoscape, and the expected level of variation between individuals growing their feathers at the same locality , . We used the standard deviation of the residuals (SD = 12.78) from our calibration equation (above) as an estimate of the expected level of variation between individuals growing their feathers at the same locality. These likelihoods were mapped one individual at a time, and were subsequently divided by the sum of all cells in the isoscape to ensure that the sum of all values in the map summed to one, yielding one ‘probability of origin’ map per sample. We then reclassified probability of origin maps for each sample to likely (1) versus unlikely (0) origins for a sample by determining the odds that any given assigned geographic origin was correct relative to the odds that it was incorrect. We selected cells associated with the upper 67% of probability densities and reclassified these as likely origins for the sample, consistent with 2:1 odds that a sample had truly originated from within the range defined by the odds ratio. This resulted in resulting in one binary map per individual. We then summed of all of the individual assignments by addition of the surfaces for each individual in the sample.

## References

Figure S1. Likelihood based assignment of house martins sampled at Gaast, The Netherlands to the *δ*^2^H isoscape of based on *δ*^2^H analysis of feathers grown in Africa in 2005 (n= 21), 2006 (n= 27), 2007 (n=71), 2008 (n= 66), 2009 (n= 84), and 2010 (n= 51). Red ellipses represent the approximate location of putative origins for house martins breeding in Italy based on correlation between winter Normalized Difference Vegetation Index and breeding ground population indices as reported by .
